# Characteristics and Phylogenetic Considerations of the Newly Sequenced Mitochondrial Genome of *Teratoscincus scincus* (Gekkota: Sphaerodactylidae)

**DOI:** 10.3390/biology15020185

**Published:** 2026-01-19

**Authors:** Zhiqiang Ge, Zhengyu Zhang, Zelu Mu, Linqiang Zhong

**Affiliations:** Xinjiang Key Laboratory of Biological Resources and Genetic Engineering, College of Life Science and Technology, Xinjiang University, Urumqi 830049, China; 107552301046@stu.xju.edu.cn (Z.G.); james_cotton@163.com (Z.Z.); 107552301054@stu.xju.edu.cn (Z.M.)

**Keywords:** *Teratoscincus scincus*, Sphaerodactylidae, mitochondrial genome, gene annotation, structural characteristics, phylogenetic analysis

## Abstract

Sphaerodactylidae play a crucial role in ecosystems, possessing significant ecological, scientific, and conservation value. They contribute to pest control and the maintenance of ecological balance, and also provide abundant materials for research in evolutionary biology and biodiversity. To refine the phylogenetic position of *Teratoscincus scincus* within the Sphaerodactylidae using mitogenomic data, this study sequenced the complete mitochondrial genome of *T. scincus* using the Illumina NovaSeq Xplus platform, and subsequently performed assembly, annotation, and analysis. The phylogenetic relationships of *T. scincus* within the Sphaerodactylidae were analyzed using 13 protein-coding genes (PCGs) from the mitochondrial genome via Bayesian inference (BI) and maximum likelihood (ML) methods. The complete mitochondrial genome of *T. scincus* is 16,943 bp in length and consists of 13 PCGs, 22 tRNA genes, 2 rRNA genes, and 1 control region (D-loop). The base composition shows a distinct AT preference, with the highest A + T content (56.3%) found in the PCGs region. A phylogenetic tree was constructed based on the amino acid sequences of 13 PCGs from the mitochondrial genomes of nine Sphaerodactylidae species retrieved from GenBank and the newly sequenced *T. scincus* generated in this study. The results confirm that *T. scincus* belongs to the genus *Teratoscincus* within the family Sphaerodactylidae. Phylogenetic analysis reveals that *T. scincus* and *Teratoscincus keyserlingii* cluster into a monophyletic group, suggesting a close phylogenetic relationship. Additionally, the phylogenetic tree provides new molecular evidence for understanding the formation mechanism of Sphaerodactylidae diversity. This study not only enriches the mitochondrial genome database of Sphaerodactylidae but also lays an important foundation for subsequent research on the adaptive evolution and conservation biology of *T. scincus*.

## Simple Summary

This study sequenced and analyzed the complete mitochondrial genome of *Teratoscincus scincus* using the Illumina NovaSeq Xplus platform. The circular mitogenome is 16,943 bp in length, comprising 13 protein-coding genes (PCGs), 22 tRNA genes, 2 rRNA genes, and 1 control region. It exhibits a distinct AT preference, with the highest A + T content (56.3%) observed in the PCGs. Phylogenetic analysis based on 13 PCGs from 10 Sphaerodactylidae species confirmed that *T. scincus* belongs to the genus *Teratoscincus*, forming a monophyletic clade with *Teratoscincus keyserlingii*. This work enriches the mitochondrial genomic database for Sphaerodactylidae and lays a foundation for research on the adaptive evolution and conservation of *T. scincus*.

## 1. Introduction

The Sphaerodactylidae, belonging to the Squamata, are a group of small to micro-sized geckos. Currently, this family is known to include 12 genera and over 200 species [1]. This group is characterized by slender body shapes, specialized ecological niches, and a wide distribution in tropical and subtropical regions, mainly concentrated in the Americas and the Caribbean; however, only a few lineages (e.g., *Teratoscincus*) have dispersed to the arid regions of Central Asia [2,3].

*Teratoscincus* is a representative genus of the Sphaerodactylidae in the arid and semi-arid regions of Central Asia and the Middle East [4]. This genus was initially classified under the Gekkonidae but was later reclassified into the Sphaerodactylidae based on molecular, morphological, and anatomical evidence [5,6]. Currently, nine species are recognized in this genus [4], three of which are distributed in China: *Teratoscincus roborowskii*, *Teratoscincus przewalskii*, and *Teratoscincus scincus* [3]. The distribution range of *T. scincus* is highly fragmented, and habitat degradation is increasingly severe; in border areas, in particular, the fragmentation of conservation policies further increases its survival risks [7].

The mitochondrial genome (mtDNA) is an essential genetic material in cells [8], exhibiting unique genetic characteristics such as maternal inheritance, a high mutation rate, and gene rearrangement [9]. It has become an ideal model for studying population genetic structure, molecular evolutionary processes, and interspecific genetic relationships in animals [10]. In most metazoans, the mitochondrial genome is a small closed circular double-stranded DNA molecule, usually containing 13 PCGs, 22 tRNA genes, and 2 rRNA genes [11,12]. In the early stage of analyzing the composition and structural characteristics of mitochondrial genomes, research mainly relied on Sanger sequencing technology [13]; in recent years, with the development of bioinformatics technology, the assembly, annotation, and analysis of mitochondrial genome sequences have been significantly improved in terms of accuracy and efficiency [14]. Molecular phylogenetic analysis based on mitochondrial genes effectively compensates for the limitations of traditional morphological classification in the identification of closely related species. Compared with phylogenetic analysis based on single genes, the multi-gene combined analysis strategy can provide more abundant genetic information and has thus become the mainstream method in phylogenetic relationship reconstruction [15].

However, there remains a significant gap in our understanding of the mitochondrial genomes of Sphaerodactylidae, particularly the arid-adapted genus *Teratoscincus*. Although *T. scincus* is one of the species distributed in China, its complete mitochondrial genome has not yet been sequenced, which has hindered in-depth phylogenetic and adaptive evolution studies on both the genus and the entire family. To fill this data gap, in this study, we sequenced, annotated, and analyzed the structural characteristics of the mitochondrial genome of *T. scincus* and constructed a phylogenetic tree. This work not only enriches the mitochondrial genome database of Sphaerodactylidae but also provides important references for the taxonomic and evolutionary research of species in this family. Furthermore, it offers a theoretical basis and data support for species diversity conservation and germplasm resource evaluation.

## 2. Materials and Methods

### 2.1. Sample Collection

A single *T. scincus* sample was collected from Huocheng County, Xinjiang Uygur Autonomous Region, China (Table 1). This sample was a roadkill carcass obtained during animal surveys. For preliminary morphological identification, we followed the key taxonomic characters of the genus *Teratoscincus* and species-specific diagnostic traits: cycloid scales of the back are strongly imbricate, extending onto the posterior part of the head, with 28–34 scales around the midbody [16]. After confirming the sample’s taxonomic identity based on these morphological characters, tail tissue was collected, stored in a 1.5 mL centrifuge tube, and preserved in a −80 °C ultra-low temperature freezer for subsequent use.

### 2.2. DNA Extraction

Genomic DNA was extracted from muscle tissue using the Blood/Cell/Tissue Genomic DNA Extraction Kit (TIANamp Genomic DNA Kit) purchased from TIANGEN BIOTECH (Beijing, China) Co., Ltd, following the manufacturer’s instructions and stored in a −20 °C freezer. To verify the accuracy of morphological identification, the mitochondrial *cox1* gene of *T. scincus* was amplified using the primers LOC1490 (5′-GGTCACAACAAATCATAAAGATATTGG-3′) and HOC2198 (5′-TAAACTTCAGGGTGACCAAAAAATCA-3′) [17]. The PCR system (25 μL) consisted of 12.5 μL of PreMix Taq enzyme, 0.5 μL of each primer (forward and reverse), 5 μL of the DNA template, and 6.5 μL of double-distilled water (ddH2O) [18]. The specific PCR conditions for the *cox1* gene are shown in Table 2. The PCR product was sent to Sangon Biotech (Shanghai, China) Co., Ltd. for Sanger sequencing. The sequencing results were subjected to BLAST v2.17.0 comparison through NCBI, and the comparison results confirmed the species as *T. scincus*.

### 2.3. Mitochondrial Genome Sequencing and Data Filtering

The muscle tissue sample of *T. scincus* was sent to Beijing Berry Genomics Co., Ltd. (Beijing, China). A genomic DNA library with an insert size of approximately 350 bp was constructed using the Illumina NovaSeq Xplus platform (Illumina, San Diego, CA, USA), and paired-end 150 bp (PE150) sequencing was performed to obtain approximately 6 GB of raw data (in FASTQ format). Subsequently, fastp v0.23.4 [19] with parameters -f 5 -t 5 -F 5 -T 5 -5 -3 -W 4 -M 20 -c -q 15 -u 40 -l 35 was used for quality control and filtering of raw reads to remove reads containing adapter sequences, high repetition rates, high N content, and low quality, thereby obtaining high-quality data for subsequent assembly.

### 2.4. Mitochondrial Genome Assembly, Annotation, and Validation

To ensure the accuracy of results, multiple strategies were adopted for the assembly and validation of the mitochondrial genome in this study. First, MitoZ v3.6 [20] was used for the integrated processing of raw sequencing data, including quality control, de novo assembly, and preliminary annotation, with the following key parameters: --clade Chordata, --genetic_code 2, --fastq_read_length 151, --data_size_for_mt_assembly 0, --assembler megahit (configured with k-mer sizes of 71 and 99), --memory 50, and --requiring_taxa Chordata. For cross-validation, high-quality reads filtered by fastp were imported into Geneious Prime v2025.1.2, and the “Map to Reference” function was used for secondary assembly. Then, the two assembly results were aligned and manually checked in MEGA v11 [21] to finally obtain a consistent high-quality mitochondrial genome sequence. Meanwhile, MITOS2 v2.1.9 [22] and tRNAscan-SE v2.0 [23] were used for gene prediction and tRNA secondary structure prediction of the *T. scincus* mitochondrial genome, respectively. The annotation results from MitoZ v3.6 and MITOS2 v2.1.9 were manually verified in Geneious Prime for final confirmation. Based on the verified genome sequence, a circular map of the mitochondrial genome was generated using the Proksee platform [24]. The final assembled sequence and annotation information have been submitted to the NCBI GenBank database with the accession number PV780393.

### 2.5. Sequence Analysis

To analyze the sequence characteristics of the mitochondrial genome, first, MEGA v11 [21] software was used to calculate the base content of the entire genome, and the nucleotide skewness was calculated according to the following skewness formulas: “AT-skew = (A − T)/(A + T)” and “GC-skew = (G − C)/(G + C)” [25]. Second, DNAsp v6.0 software was used to conduct an in-depth analysis of the evolutionary characteristics of the genome using the *Ka/Ks* ratio (non-synonymous/synonymous substitution rate ratio) as an indicator. In addition, PhyloSuite v1.2.3 [26,27] software was used to calculate the relative synonymous codon usage (RSCU) of PCGs, and the results of codon usage analysis were visualized.

### 2.6. Phylogenetic Analysis

Based on Bayesian inference and maximum likelihood methods, the effective mitochondrial genomes of 10 Sphaerodactylidae species and the mitochondrial genome of *Eublepharis macularius* (NC033383) as the outgroup were selected to conduct phylogenetic analysis of their 13 PCGs (Table 3). All analyses were completed on the PhyloSuite v1.2.3 [26,27] platform. The specific workflow was as follows: First, the corresponding GenBank format sequences were downloaded from the NCBI database and imported into PhyloSuite v1.2.3 for gene screening and annotation. MAFFT v7.526 was used for multiple sequence alignment [28]; then, MACSE v2 [29] was used to optimize the alignment results, followed by trimming with trimAl v2 [30] to obtain optimized aligned sequences. FASconCAT-G v1.04 [31] was used to concatenate the sequences. IQ-TREE v2.2.0 [32] was used to construct the ML tree. Under the Akaike information criterion, ModelFinder v2.2.0 was used to determine the optimal partitioning scheme (edge-linked) and substitution model, and 1000 bootstrap replicates were used to evaluate the confidence values of each node in the phylogenetic tree [33]. MrBayes v3.2.7 [34] with 2 independent runs was used to construct the BI tree, with each run having 4 Markov chain Monte Carlo (MCMC) chains of 5,000,000 generations, sampled every 1000 generations. The first 25% of the runs were discarded to estimate the posterior probabilities of branch confidence. ModelFinder v2.2.0 and the corrected Bayesian information criterion were used to determine the optimal partitioning scheme (edge-linked) and model. Finally, the online software ChiPlot v1 was used to visualize and refine the phylogenetic tree [35].

## 3. Results

### 3.1. Genome Organization and Base Composition

**Figure 1:**
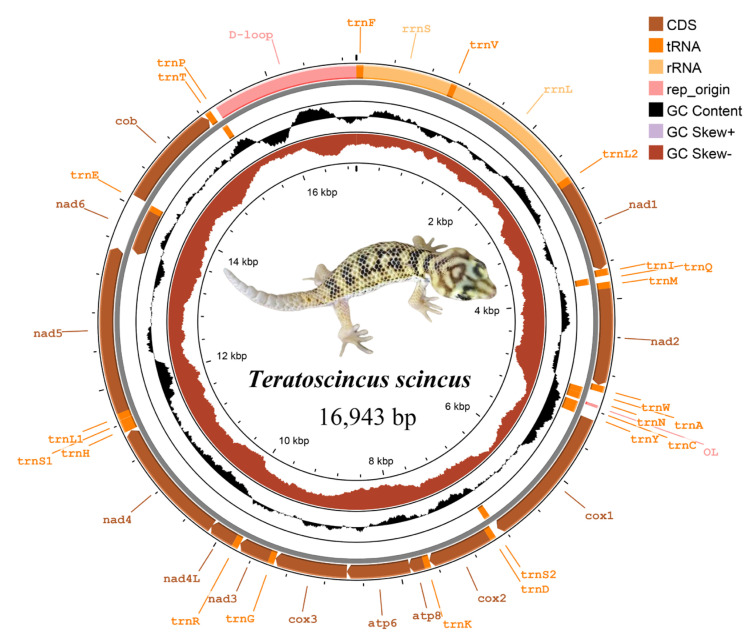
Mitochondrial genome structure of *T. scincus*.

The complete mitochondrial genome of *T. scincus* obtained via sequencing is a typical double-stranded circular DNA molecule with a size of 16,943 bp (Figure 1). The mitochondrial genome of *T. scincus* sequenced in this study contains 13 PCGs, 22 transfer RNA (tRNA) genes, 2 ribosomal RNA (rRNA) genes, and 1 non-coding sequence (D-loop region). Eight tRNA genes (*trnQ*, *trnA*, *trnN*, *trnC*, *trnY*, *trnS2*, *trnE*, *trnP*) and *nad6* are located on the L-strand, while the remaining genes are on the H-strand (Table 4). There are eight gene overlaps and six gene intervals in the mitochondrial genome (Table 4).

The gene overlaps are located between *trnI* and *trnQ* (−1 bp), *trnQ* and *trnM* (−1 bp), *trnW* and *trnA* (−1 bp), OL and *trnC* (−2 bp), *cox1* and *trnS2* (−9 bp), *atp8* and *atp6* (−10 bp), *nad4L* and *nad4* (−7 bp), and *nad5* and *nad6* (−8 bp), with the longest sequence being 10 bp and the shortest 1 bp. The gene intervals are located between *trnA* and *trnN* (1 bp), *trnY* and *cox1* (1 bp), *trnR* and *nad4L* (1 bp), *nad4* and *trnH* (5 bp), *trnS1* and *trnL1* (2 bp), and *trnE* and *cob* (3 bp), with the longest sequence being 5 bp and the shortest 1 bp.

In the complete mitochondrial genome of *T. scincus*, the contents of bases A, T, G, and C are 30.2%, 25.6%, 14.5%, and 29.7%, respectively, and the content of A + T (55.8%) is significantly higher than that of C + G (44.2%). Nucleotide composition analysis showed that *T. scincus* has a significant preference for AT nucleotides, i.e., there is AT bias in the mitochondrial genome. The AT-skew (0.082) is greater than the GC-skew (−0.345), and this pattern is also evident in the PCGs (AT-skew: 0.055 > GC-skew: −0.406), tRNAs (AT-skew: 0.110 > GC-skew: −0.204), and rRNAs (AT-skew: 0.230 > GC-skew: −0.221). The A + T content of PCGs in the mitochondrial genome is the highest (56.3%) (Table 5).

### 3.2. Protein-Coding Genes and Codon Usage Patterns

The total length of the 13 PCGs in the *T. scincus* mitochondrial genome is 11,367 bp (Table 5). Except for *nad6*, which is encoded on the L-strand, the others are encoded on the H-strand (Table 4). Among the PCGs of the mitochondrial genome, *atp8* is the shortest (165 bp) and *nad5* is the longest (1803 bp) (Table 4). In addition, *nad1* starts with the ATC codon, *nad2* with ATA, *nad3* with ATT, *nad5*, *cox1*, and *atp8* with GTG, and the remaining start codons are ATG (Table 4).

**Figure 2:**
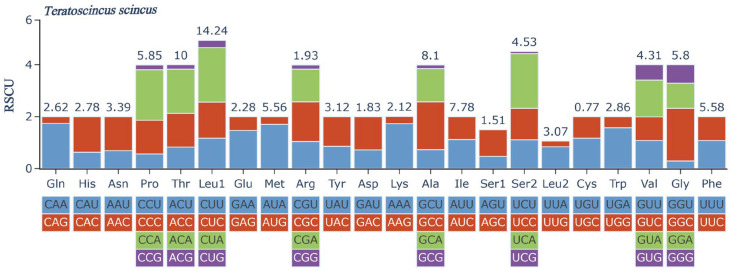
Amino acid usage and relative synonymous codon usage of protein-coding genes in the *T. scincus* mitochondrial genome.

Five types of stop codons were identified in the PCGs of *T. scincus*, including incomplete codons T and TA, and complete codons TAA, TAG, and AGG (Table 4). *nad2* and *cox3* use T as the stop codon, *cox1* uses AGG, *atp6*, *cob*, and *nad3* use TA, *nad1*, *nad4L*, *nad4*, *nad6*, and *atp8* use TAA, and *nad5* uses TAG (Table 4). Among them, the TAA stop codon has the highest frequency of occurrence, while TAG and AGG have the lowest (Table 4). Through RSCU analysis, we studied the codon usage pattern of the *T. scincus* mitochondrial genome (Figure 2). The RSCU value of codon UCA is the highest (2.11), and that of UCG is the lowest (0.08) (Table 6).

### 3.3. Transfer RNAs, Ribosomal RNAs, and Control Region

**Figure 3:**
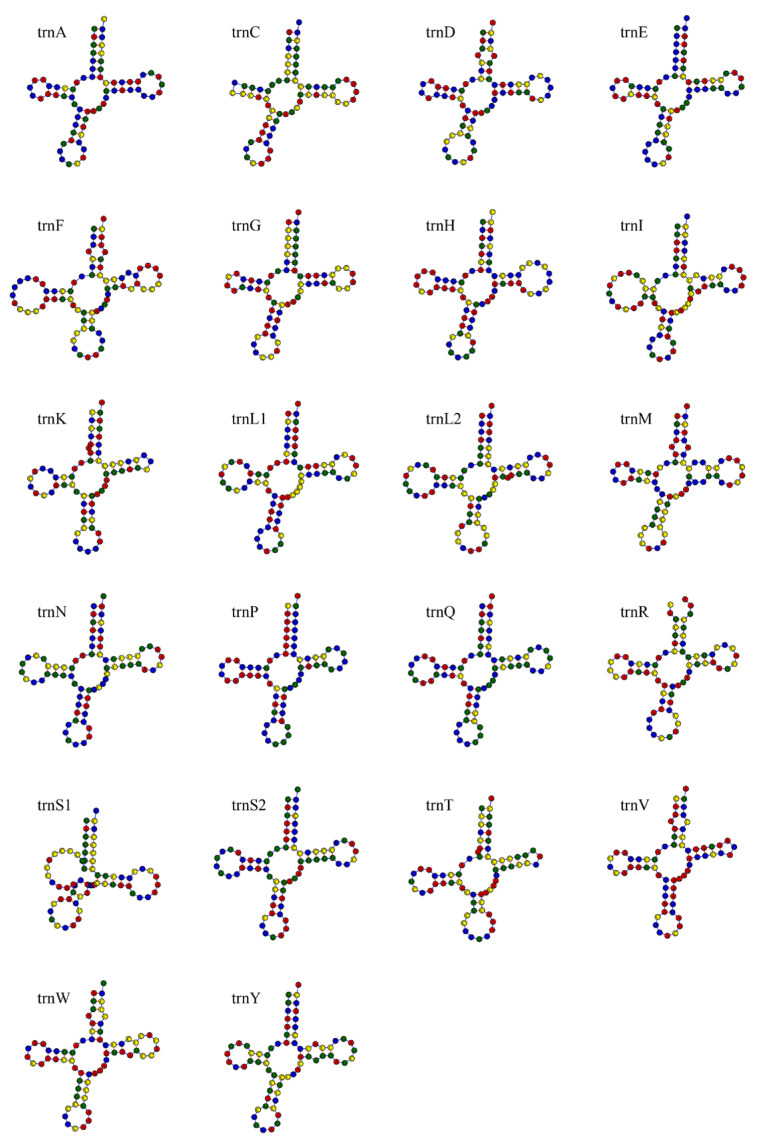
Secondary structures of tRNAs in the mitochondrial genome of *T. scincus*. Red indicates adenine (A), yellow indicates cytosine (C), blue indicates uracil (U), and green indicates guanine (G).

A total of 22 tRNA genes were identified in the *T. scincus* mitochondrial genome, with each gene ranging in length from 66 to 75 bp, totaling 1532 bp. Fourteen tRNA genes are located on the H-strand, and eight on the L-strand. Among them, 21 tRNA genes can fold into the typical cloverleaf secondary structure, but the DHU arm of *trnS1* is missing and fails to form a typical stem-loop structure (Figure 3).

**Figure 4:**
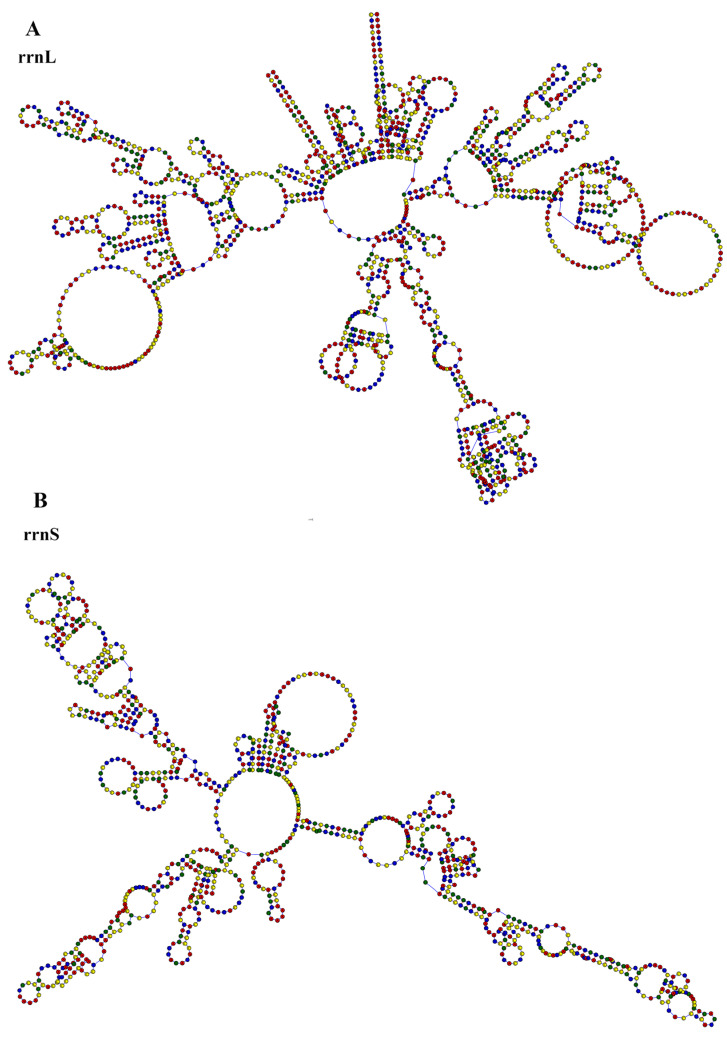
Secondary structures of rRNAs in the mitochondrial genome of *T. scincus.* Secondary structure of the large subunit ribosomal RNA (*rrnL*) (**A**); Secondary structure of the small subunit ribosomal RNA (*rrnS*) (**B**). Red indicates adenine (A), yellow indicates cytosine (C), blue indicates uracil (U), and green indicates guanine (G).

The *T. scincus* mitochondrial genome contains a typical ribosomal RNA (rRNA) gene cluster, consisting of *rrnL* (1524 bp) (Figure 4A) and *rrnS* (949 bp) (Figure 4B), with a total length of 2473 bp. These two rRNA genes are located between the *trnF* (75 bp) and *trnL2* (75 bp) genes, separated by the *trnV* (66 bp) gene. OL is located between the *trnN* and *trnC* genes, with a length of 31 bp (Table 4); the D-loop region is located between the *trnP* and *trnF* genes, with a length of 1566 bp (Table 4).

### 3.4. Synonymous and Non-Synonymous Substitution Rates

**Figure 5:**
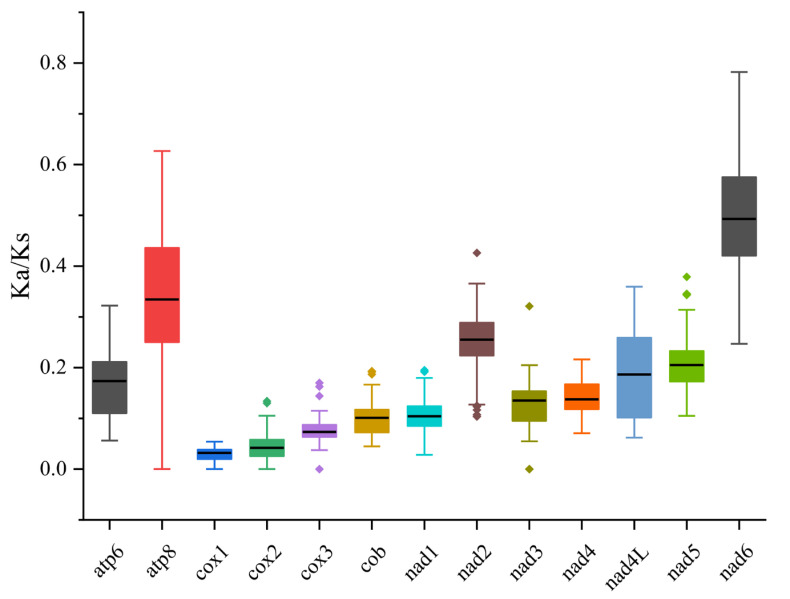
*Ka/Ks* values of 13 protein-coding genes of Sphaerodactylidae mitochondrial genomes. The solid black line indicates the mean value.

To study the evolutionary patterns and rates of mitochondrial PCGs in Sphaerodactylidae species, this study calculated the *Ka*/*Ks* ratios of homologous gene pairs of mitochondrial PCGs from 10 Sphaerodactylidae species and analyzed them in combination with the *Ka/Ks* values (Figure 5). The results showed that the average *Ka/Ks* ratios of the 13 PCGs in descending order are *nad6*, *atp8*, *nad2*, *nad5*, *nad4L*, *atp6*, *nad4*, *nad3*, *nad1*, *cob*, *cox3*, *cox2* and *cox1*. The *Ka/Ks* ratio for all 13 PCGs was found to be below 1.

### 3.5. Phylogenetic Relationships

Using *E. macularius* (NC033383) as the outgroup, combined with the gene sequence of *T. scincus* (PV780393) sequenced in this study, phylogenetic analysis was conducted on the 13 PCG sequences of 9 Sphaerodactylidae species published in GenBank (Table 3). The phylogenetic tree showed that all *Teratoscincus* species clustered into a monophyletic group with high support, confirming the taxonomic status of this genus within Sphaerodactylidae. Within this genus, *T. scincus* and *Teratoscincus keyserlingii* clustered into one clade with high support (BI/ML > 99%), while *T. przewalskii* and *T. roborowskii* formed another monophyletic group, and *Teratoscincus microlepis* was the sister group of the above four species.

## 4. Discussion

In this study, we described and analyzed the complete mitochondrial genome of *T. scincus*, and examined its structural features, evolutionary patterns, and phylogenetic position within Sphaerodactylidae. It has a typical vertebrate structure with a length of 16,943 bp (13 PCGs, 22 tRNAs, 2 rRNAs, 1 D-loop) [7,36]. This work fills an existing gap in mitochondrial genomic data for this species and provides a useful reference for comparative mitogenomic and phylogenetic studies within the family.

Eight gene overlaps were observed in the *T. scincus* mitochondrial genome, among which the overlap region between *atp8* and *atp6* is the largest. This may facilitate the co-transcription and coordinated expression of these two functionally related genes, reflecting the economy of the mitochondrial genome [11,37]. Gene overlap not only saves bases and efficiently uses DNA but also may participate in gene regulation, allowing limited DNA to carry more genetic information, which reflects the rational use of genetic material by organisms [38]. This pattern may be consistent with adaptations to energy constraints in arid environments where resources are scarce, although direct functional tests are required.

The *T. scincus* mitochondrial genome shows a significant AT bias (A + T = 55.8%), with the highest AT content (56.3%) in the PCGs region (Table 5). This bias originates from the asymmetric replication of mitochondria [39], which can reduce the stability of DNA double strands and accelerate mutation accumulation, providing a genetic basis for the rapid generation of adaptive variations in arid environments [40,41].

The start and stop codon usage patterns of PCGs in *T. scincus* have certain particularities, and these variations may affect their transcription and translation efficiency, thereby playing a role in the evolutionary process [42,43,44]. Studies have found that in reptiles, ATG is the most common start codon, and some genes use incomplete stop codons (e.g., T and TA), which may be related to their living environment and physiological needs. Incomplete stop codons (e.g., T and TA) may complete the termination function through polyadenylation. Meanwhile, codon usage patterns can be used to distinguish different groups, and the frequent use of codons A and T with an obvious AT skew may be related to the compositional asymmetry of the mitochondrial genome, which also provides a genetic basis for *T. scincus* to rapidly generate adaptive variations in response to changes in arid environments [45].

The transcription of *T. scincus* mitochondrial genes is mainly based on the H-strand, while some genes are transcribed by the L-strand. This double-strand transcription mode ensures the efficient expression of mitochondrial genes [46]. tRNA genes are asymmetrically distributed on the two strands, with 14 tRNA genes located on the heavy strand (H-strand) and 8 on the light strand (L-strand). This distribution pattern is consistent with the typical characteristics of vertebrate mitochondrial genomes, ensuring the efficient transcription and translation of mitochondrial genes [47]. Twenty-one tRNA genes can fold into the typical cloverleaf secondary structure, which is crucial for maintaining the normal function of the mitochondrial protein synthesis system [47]. The genome shows a typical gene arrangement pattern of *trnF*/*rrnS*/*trnV*/*rrnL*/*trnL2*, which is widely present in vertebrate mitochondrial genomes [11]. The structural characteristics of rRNAs in *T. scincus* (the secondary structures of *rrnS* and *rrnL* are composed of multiple stem-loops, and *rrnL* is more complex than *rrnS*) may reflect adaptive changes during its evolution [48]. The non-coding regions include OL and D-loop. Among them, OL mainly functions to initiate the replication of the L-strand, while the D-loop is used to regulate transcription and initiate the replication of the H-strand. The two cooperate to maintain the stable replication and expression of the mitochondrial genome [49].

The adaptive characteristics of Sphaerodactylidae in morphology, behavior, and molecular levels are closely related to environmental pressures [3]. *Nad6 is* a core subunit of mitochondrial respiratory chain complex I (NADH dehydrogenase), whose core function is to participate in electron transfer and energy generation in cellular aerobic respiration [50]. The results suggest that nad6 is a fast-evolving gene, potentially driven by adaptive selection or relaxed constraints [51]. In extreme environments with scarce resources and high environmental pressure, species need to efficiently use limited energy to maintain survival [52]. These findings suggest that *nad6* may contribute to environmental adaptation mechanisms in Squamate reptiles [53]. This is crucial for *T. scincus* to resolve the contradiction between "low food intake and high energy demand" in arid regions and can help it efficiently synthesize ATP under limited resources to maintain energy supply for nocturnal activities (such as predation and enemy avoidance).

**Figure 6:**
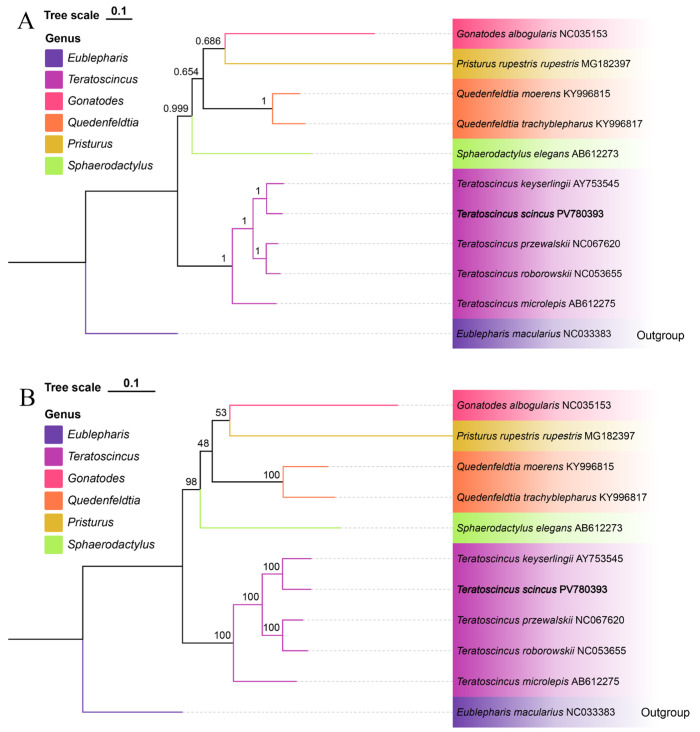
The Bayesian inference (BI) phylogenetic tree (**A**) and maximum likelihood (ML) phylogenetic tree (**B**) were constructed based on the amino acid sequences of 13 protein-coding genes from 10 Sphaerodactylidae species (including the newly sequenced *T. scincus*) and one outgroup species (*E. macularius*, Eublepharidae).

Phylogenetic analysis showed that *T. scincus* and *T. keyserlingii* clustered into a monophyletic group with high support (BI/ML support > 99%). Moreover, *T. przewalskii* and *T. roborowskii* formed another monophyletic group, and *T. microlepis* was the sister group of the two (Figure 6). This clade division is highly consistent with geographical distribution: the former clade is distributed in the arid regions of Central Asia (including Huocheng, Xinjiang and Iran), and the latter is restricted to the arid regions of northwestern China (Tarim Basin, Turpan Basin), indicating that the geographical isolation effect of the Tian Shan Mountains may play a key role in the species differentiation of the genus *Teratoscincus* [54]. These results not only confirm the taxonomic status of *T. scincus* within Sphaerodactylidae but also provide molecular evidence for the "geographical clade" division of the genus *Teratoscincus*, which can be used to revise the taxonomic system of this genus.

This study has two limitations: first, the phylogenetic analysis only included 10 Sphaerodactylidae species and did not cover all 9 species of the genus *Teratoscincus*, which may underestimate the deep differentiation within the genus; second, only mitochondrial gene data were used, and nuclear genes were not combined to verify the phylogenetic relationship, making it difficult to exclude the maternal inheritance bias of mitochondrial genes. In the future, it is necessary to supplement *Teratoscincus* species samples across geographical regions and integrate mitochondrial and nuclear gene data. This will not only clarify the evolutionary history of this genus but also further locate key nuclear gene loci for arid adaptation.

## 5. Conclusions

Through this study, we successfully determined, assembled, and annotated the complete mitochondrial genome of *T. scincus* (16,943 bp). A phylogenetic tree was constructed by combining the mitochondrial genomes of nine Sphaerodactylidae species (with *E macularius* as the outgroup). The results showed the following: The *T. scincus* mitochondrial genome has a typical vertebrate structure with a significant AT bias (A + T = 55.8%). And Phylogenetic analysis supports that *T. scincus* and *T. keyserlingii*, as well as *T. przewalskii* and *T. roborowskii* form sister groups, respectively, which confirms the driving effect of geographical isolation imposed by the Tian Shan Mountains on species differentiation within the genus *Teratoscincus*. This study provides basic data for the taxonomic revision of Sphaerodactylidae and the conservation genetics research of *T. scincus*.

## Figures and Tables

**Table 1:** Details of samples used in this study.

Species	Sample ID	Collection Date	Gender	Collection Site	Altitude	Samples
*Teratoscincus scincus*	XJU-1	25 May 2024	Female	Huocheng County, Xinjiang Uygur Autonomous Region, China (80.783124° E, 43.973877° N)	752 m	Tail tissue

**Table 2:** PCR reaction conditions for *cox1*.

PCR Reaction Program	Temperature (°C)	Cycle Number	Time (s)
Initial denaturation	94	1	60
Denaturation	94	6	60
Annealing	45	6	90
Elongation	72	6	75
Denaturation	94	36	60
Annealing	51	36	60
Elongation	72	36	75
Final elongation	72	1	600
Storage	4	∞	∞

*Note*: The symbol “∞” represents indefinite storage at 4 °C.

**Table 3:** GenBank accession numbers of species sampled in the present study.

No	Family	Genus	Species	Accession	Length (bp)	AT%
1	Sphaerodactylidae	*Gonatodes*	*Gonatodes albogularis*	NC035153	16,830	52.0
2	Sphaerodactylidae	*Pristurus*	*Pristurus rupestris rupestris*	MG182397	16,993	52.1
3	Sphaerodactylidae	*Quedenfeldtia*	*Quedenfeldtia moerens*	KY996815	16,455	51.8
4	Sphaerodactylidae	*Quedenfeldtia*	*Quedenfeldtia trachyblepharus*	KY996817	17,060	51.3
5	Sphaerodactylidae	*Sphaerodactylus*	*Sphaerodactylus elegans*	AB612273	17,500	54.2
6	Sphaerodactylidae	*Teratoscincus*	*Teratoscincus keyserlingii*	AY753545	18,400	56.5
7	Sphaerodactylidae	*Teratoscincus*	*Teratoscincus microlepis*	AB612275	16,995	54.2
8	Sphaerodactylidae	*Teratoscincus*	*Teratoscincus przewalskii*	NC067620	17,184	55.8
9	Sphaerodactylidae	*Teratoscincus*	*Teratoscincus roborowskii*	NC053655	16,693	56.2
**10**	**Sphaerodactylidae**	***Teratoscincus***	***Teratoscincus scincus***	**PV780393**	**16,943**	**55.8**
11	Eublepharidae	*Eublepharis*	*Eublepharis macularius*	NC033383	17,462	63.1

*Note*: Bold text specifically refers to the species information sequenced in this study.

**Table 4:** Structure of the mitochondrial genome of *T. scincus*.

	Position		Gene	Intergenic		Codon		
Gene	From	To	Length (bp)	Nucleotide (bp)	Anticodon	Start	Stop	Strand
*trnF*	1	75	75	0	GAA	-	-	H
*rrnS*	76	1024	949	0	TAC	-	-	H
*trnV*	1025	1090	66	0	-	-	-	H
*rrnL*	1091	2614	1524	0	-	-	-	H
*trnL2*	2615	2689	75	0	TAA	-	-	H
*nad1*	2690	3667	978	0	-	ATC	TAA	H
*trnI*	3668	3740	73	0	GAT	-	-	H
*trnQ*	3740	3811	72	−1	TTG	-	-	L
*trnM*	3811	3880	70	−1	CAT	-	-	H
*nad2*	3881	4925	1045	0	-	ATA	T--	H
*trnW*	4926	4994	69	0	TCA	-	-	H
*trnA*	4994	5062	69	−1	TGC	-	-	L
*trnN*	5064	5136	73	1	GTT	-	-	L
OL	5137	5167	31	0	-	-	-	-
*trnC*	5166	5231	66	−2	GCA	-	-	L
*trnY*	5232	5300	69	0	GTA	-	-	L
*cox1*	5302	6858	1557	1	-	GTG	AGG	H
*trnS2*	6850	6921	72	−9	TGA	-	-	L
*trnD*	6922	6989	68	0	GTC	-	-	H
*cox2*	6990	7674	685	0	-	ATG	T--	H
*trnK*	7675	7741	67	0	TTT	-	-	H
*atp8*	7742	7906	165	0	-	GTG	TAA	H
*atp6*	7897	8576	680	−10	-	ATG	TA-	H
*cox3*	8577	9360	784	0	-	ATG	T--	H
*trnG*	9361	9427	67	0	TCC	-	-	H
*nad3*	9428	9774	347	0	-	ATT	TA-	H
*trnR*	9775	9842	68	0	TCG	-	-	H
*nad4L*	9844	10,140	297	1	-	ATG	TAA	H
*nad4*	10,134	11,498	1365	−7	-	ATG	TAA	H
*trnH*	11,504	11,574	71	5	GTG	-	-	H
*trnS1*	11,575	11,641	67	0	GCT	-	-	H
*trnL1*	11,644	11,715	72	2	TAG	-	-	H
*nad5*	11,716	13,518	1803	0	-	GTG	TAG	H
*nad6*	13,511	14,032	522	−8	-	ATG	TAA	L
*trnE*	14,033	14,100	68	0	TTC	-	-	L
*cob*	14,104	15,242	1139	3	-	ATG	TA-	H
*trnT*	15,243	15,310	68	0	TGT	-	-	H
*trnP*	15,311	15,377	67	0	TGG	-	-	L
D-loop	15,378	16,943	1566	0	-	-	-	-

**Table 5:** Nucleotide composition of the mitochondrial genome of *T. scincus*.

Region	Length (bp)	A	C	G	T	G + C%	A + T%	GC-Skew	AT-Skew
Total genome	16,943	30.2	29.7	14.5	25.6	44.2	55.8	−0.345	0.082
PCGs	11,367	29.7	30.7	13.0	26.6	43.6	56.3	−0.406	0.055
tRNAs	1532	31.0	26.6	17.6	24.9	44.1	55.9	−0.204	0.110
rRNAs	2473	33.5	27.8	17.8	20.9	45.6	54.4	−0.221	0.230
D-loop	1566	28.1	28.2	16.8	26.9	45.0	55.0	−0.254	0.022

**Table 6:** Relative codon usage frequency of the mitochondrial genome of *T. scincus*.

Codon	Count	RSCU	Codon	Count	RSCU	Codon	Count	RSCU	Codon	Count	RSCU
UUU (F)	114	1.08	UCU (S)	42	1.11	UAU (Y)	51	0.86	UGU (C)	17	1.17
UUC (F)	97	0.92	UCC (S)	46	1.21	UAC (Y)	67	1.14	UGC (C)	12	0.83
UUA (L)	92	0.84	UCA (S)	80	2.11	UAA (*)	5	2.86	UGA (W)	85	1.57
UUG (L)	24	0.22	UCG (S)	3	0.08	UAG (*)	1	0.57	UGG (W)	23	0.43
CUU (L)	127	1.17	CCU (P)	31	0.56	CAU (H)	33	0.63	CGU (R)	19	1.04
CUC (L)	151	1.39	CCC (P)	72	1.3	CAC (H)	72	1.37	CGC (R)	28	1.53
CUA (L)	229	2.1	CCA (P)	108	1.95	CAA (Q)	86	1.74	CGA (R)	23	1.26
CUG (L)	31	0.28	CCG (P)	10	0.18	CAG (Q)	13	0.26	CGG (R)	3	0.16
AUU (I)	165	1.12	ACU (T)	78	0.83	AAU (N)	44	0.69	AGU (S)	18	0.47
AUC (I)	129	0.88	ACC (T)	123	1.3	AAC (N)	84	1.31	AGC (S)	39	1.03
AUA (M)	178	1.7	ACA (T)	161	1.7	AAA (K)	69	1.73	AGA (*)	0	0
AUG (M)	32	0.3	ACG (T)	16	0.17	AAG (K)	11	0.28	AGG (*)	1	0.57
GUU (V)	44	1.08	GCU (A)	56	0.73	GAU (D)	25	0.72	GGU (G)	16	0.29
GUC (V)	37	0.91	GCC (A)	141	1.84	GAC (D)	44	1.28	GGC (G)	111	2.03
GUA (V)	58	1.42	GCA (A)	98	1.28	GAA (E)	63	1.47	GGA (G)	53	0.97
GUG (V)	24	0.59	GCG (A)	11	0.14	GAG (E)	23	0.53	GGG (G)	39	0.71

*Note*: The symbol “*” represents stop codons in the mitochondrial genome of *T. scincus*.

## Data Availability

The complete mitogenomes of *T. scincus* has been published in the GenBank public database with accession number PV780393.
